# A syndrome featuring developmental disorder of the nervous system induced by a novel mutation in the *TCF20* gene, rarely concurrent immune disorders: a case report

**DOI:** 10.3389/fgene.2023.1192668

**Published:** 2023-05-25

**Authors:** Si Huang, Jiaxin Xu, Yiyang Li, Wenhui Mo, Xiuwen Lin, Yajun Wang, Fujian Liang, Yan Bai, Guochun Huang, Jing Chen, Jing Xin, Guoda Ma

**Affiliations:** ^1^Department of Pediatrics, Foshan Fosun Chancheng Hospital, Foshan, China; ^2^Department of Pediatrics, Shunde Women and Children’s Hospital of Guangdong Medical University, Foshan, China; ^3^ Institute of Maternal and Child Research, Shunde Women and Children’s Hospital of Guangdong Medical University, Foshan, China; ^4^ Key Laboratory of Research in Maternal and Child Medicine and Birth Defects, Guangdong Medical University, Foshan, China; ^5^ Department of Pediatrics, The Third Affiliated Hospital of Guangzhou Medical University, Guangzhou, China

**Keywords:** TCF20, frameshift mutation, central nervous system, immune system diseases, multisystem

## Abstract

**Background:** The expression of TCF20 is the most widespread in brain tissue. TCF20 depletion or mutation can affect the proliferation and differentiation of embryonic neurons, leading to developmental disorder of the central nervous system and subsequent rare syndrome featuring.

**Case presentation:** Here, we report a 3-year-old boy carrying a novel frameshift mutation in *TCF20,* c.1839_1872del (p.Met613IlefsTer159), resulting in multisystem disease. In addition to symptoms of neurodevelopmental disorder, a large head circumference, special appearance, overgrowth, abnormal testicular descent. Remarkably, previously infrequently reported symptoms of the immune system such as hyperimmunoglobulinemia E (hyper-IgE), immune thrombocytopenic purpura, cows milk protein allergy, and wheezy bronchitis, were observed.

**Conclusion:** This study broadens the mutation spectrum of the *TCF20* and the phenotypic spectrum of TCF20-associated disease.

## Introduction

Transcriptor co-activator factor 20 gene (*TCF20*), located on chromosome 22q13.2, encodes a nuclear chromatin-binding protein with implications for gene expression regulation. It is expressed by the majority of tissues in the body except the ovarian and prostate (Rekdal et al., 2000). Notably, the expression of TCF20 is the most widespread in brain tissue while the highest in hippocampus and cerebellum (Babbs et al., 2014; Vetrini et al., 2019; Lévy et al., 2022).

TCF20 pathogenic variants or deletions can affect the proliferation and differentiation of embryonic neurons, leading to developmental disorder of the central nervous system and subsequent rare syndrome featuring neuropsychiatric symptoms. There may be some psychobehavioural abnormalities, such as autism spectrum disorder and attention-deficit hyperactivity disorder, and symptoms of the nervous systems, such as intellectual impairment, delayed motor development, ataxia, reduced muscle strength and hypotonia, speech impairment, seizure and structural brain abnormalities. Additionally, some patients may develop concomitant congenital abnormalities of other systems, such as overgrowth, large head circumference, special appearance, and developmental malformation of the genito-urinary system, etc. (Torti et al., 2019; Vetrini et al., 2019; Lévy et al., 2022).

This report was approved by the Medical Ethics Committee of the hospital, and informed consent was obtained from the patient’s parents. Here, we report a case of multisystem involvement due to a novel frameshift mutation in the *TCF20*. In addition to symptoms of neurodevelopmental disorder (NDD), a large head circumference, special appearance, overgrowth, abnormal testicular descent. Remarkably, previously rarely reported symptoms of the immune system (e.g., hyperimmunoglobulinemia E [hyper-IgE], immune thrombocytopenic purpura, cows milk protein allergy, and wheezy bronchitis) were present.

## Case presentation

Authorized by the Medical Ethics Committee of the hospital (Approval number: LW-2023-001) and with the informed consent of the patient’s parents.

The patient was a boy. He was a second-born delivered by spontaneous vaginal birth at 38+4 weeks, and his birth weight was 3,200 g, birth length was 50 cm and birth head circumference was 35 cm. The patient had a 1-min Apgar score of 10, a 5-min Apgar score of 10, a 10-min Apgar score of 10. There was no history of birth asphyxia. At age 1 month, the Neonatal Behavioral Neurological Assessment (NBNA) score 31 was obtained (NBNA score <35 indicates possibility of having poor prognosis and intellectual disability).

**FIGURE 1:**
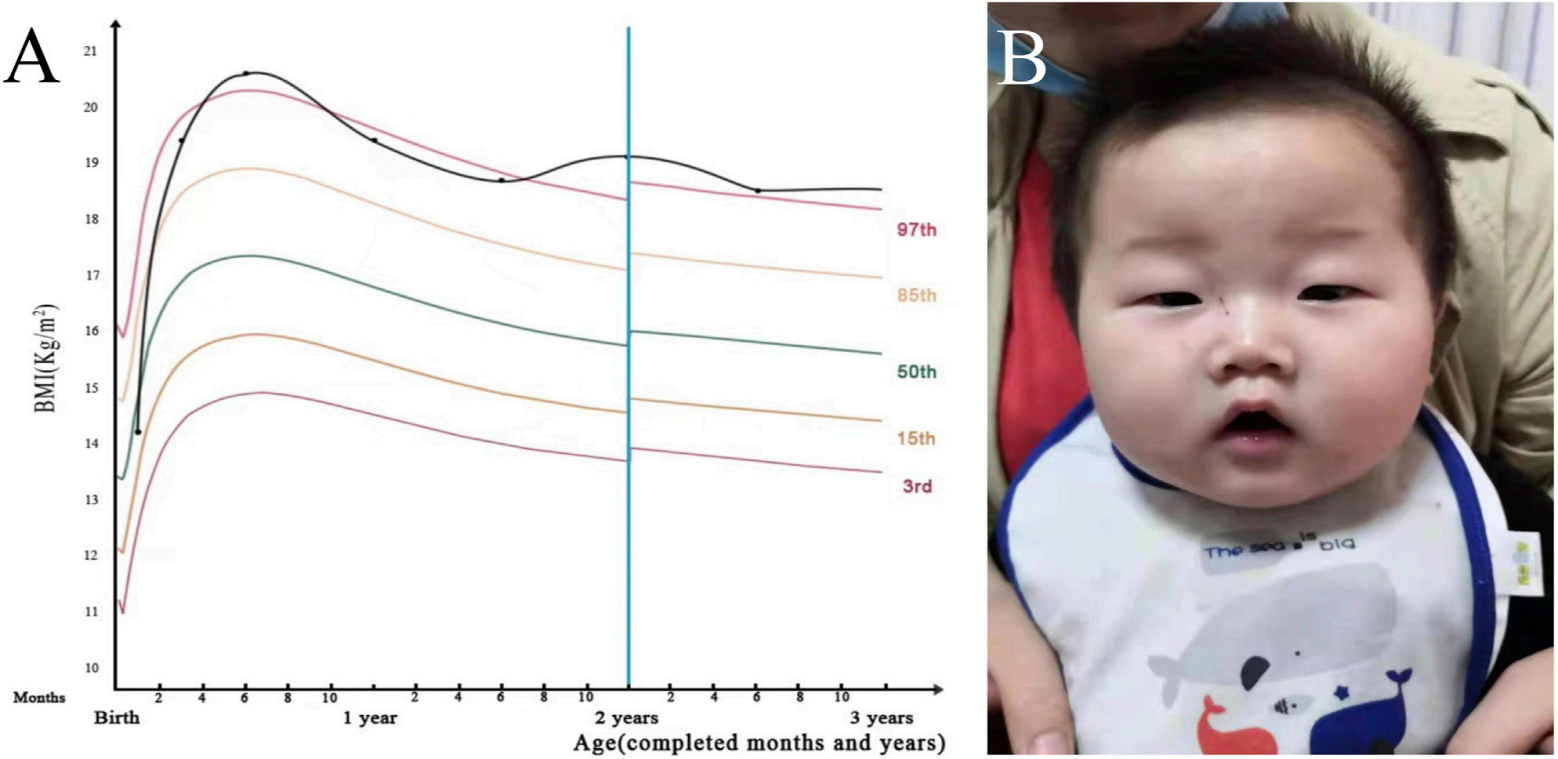
Clinical features of the patient. **(A)**. BMI growth curve of the patient. BMI = weight (kg)/height squared (m^2^). In children, BMI ranging 85th–95th of the reference for the same sex and age group indicates overweight, while BMI >95th suggests obesity. **(B)**. Characteristic appearance of the patient.

**FIGURE 2:**
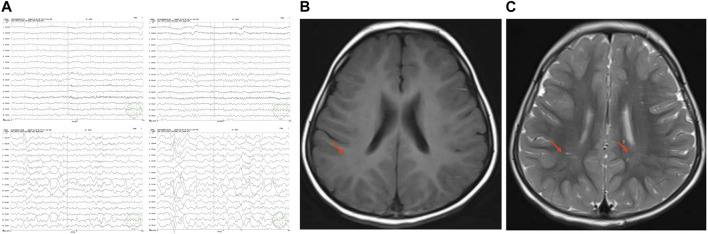
EEG and cranial MRI. **(A)**. EEG of the patient at age 7 months. Abnormal background rhythm was found. **(B–C)**. Axial T1MI and T2MI on Cranial MRI. Symmetric patchy abnormal signals in the bilateral parietal white matter and adjacent to the bilateral lateral ventricles, and insufficient cerebral white matter myelination were observed. The nuclei in the bilateral basal ganglia showed symmetric slightly short T1 and slightly short T2 signals; the bilateral lateral paraventricular region and bilateral parietal white matter revealed symmetric patchy slightly long T1 and slightly long T2 signals, isointense signals on fluid attenuated inversion recovery (FLAIR) image, and slightly low-intense signal with indistinct edges on diffusion weighted imaging (DWI).

At age 7 months, the patient was mixed-fed (breast milk and formula) and hyperphagic, therefore leading to obesity. His body weight was 11.5 kg > +3SD, body length was 71 cm [+SD - +2SD], head circumference was 45 cm > 90%, and anterior fontanel was 3 cm * 3 cm. The body mass index (BMI) exponential growth curve was shown in Figure 1A. Besides, a special appearance was presented: large head circumference, prominent forehead, plump face, short neck, small palpebral fissure, long eye distance, down-turned corners of the mouth (presenting as a triangle), (Figure 1B). Regarding motor development, the patient was unable to hold head steady while sitting, and not able to rolls over, crawls or creep-crawls. Additionally, the patient was unable to reach for and grasp large objects or transfer objects from hand to hand. The four limbs demonstrated hypotonia and grade III muscle strength. As for hearing and language development, the patient could not turn and look in the direction of sounds or respond to requests, such as “Come here,” despite normal hearing test results. The patient is unable to use speech or noncrying sounds to gain and maintain attention, as well as to imitate various speech sounds. Regarding neuropsychological and emotional development, the patient was unresponsive to changes in emotional content during social interaction and showed insensitivity to pain, as well as a lack of responsiveness toward games. The Gesell Developmental Schedules (GDS) scores were obtained from five domains: gross motor score 60, fine motor score 66.7, adaptability score 55.7, language score 65.7, and social personality score 50, with a total score of 59.6, indicating mild intellectual disability. The Alberta Infant Motor Scale (AIMS) showed a score of 15 in total (<1%), suggesting delayed motor development. Bilateral testes did not descend to the scrotum. The electroencephalogram (EEG) revealed a background rhythm which was abnormal (Figure 2A). Cranial magnetic resonance imaging (MRI) showed fullness in the lateral ventricles and widened extra-cerebral spaces. Biochemical parameters tested included cholesterol and triglycerides, which were detected as 5.86 mmol/L (0–5.18 mmol/L) and 1.93 mmol/L (1.0–1.7 mmol/L), respectively. Chromosome analysis revealed a karyotype, 46, XY, of the chromosomes of peripheral blood cells, and no abnormalities were found in chromosomal microarray analysis as well as in amino acid assay in blood and urine. The liver, gallbladder, pancreas, spleen, and urinary system were found normal on ultrasound. Brainstem auditory and visual evoked potentials were also normal.

At age 10 months, the patient could climb but was unable to stand or eat by himself. He spoke less, feared eye contact with others, showed little interest in things around him and lazy movement. The GDS showed the gross motor month 6.3, fine motor month 7.6, adaptability month 6.7, language month 6, and social personality month 6.5, and intelligence age 6.5 months, with a total score of 66.3.

At age 1 year and 6 months, the patient could walk independently, but he was easy to fall down due to a gait problem caused by hypotonia. The proband cannot communicate basic words such as “bye-bye,” “Dada,” and “Mama.”At 2 years and 3 months old, the proband exhibited reduced or absent use of eye contact in social situations, delayed or absent response to their name being called despite normal hearing, reduced or absent responsiveness to others’ facial expressions or feelings, unusually negative response to requests from others (“demand avoidance” behavior), and language delay. They struggle to combine two words together, such as “more cookie” or “no juice,” and have difficulty using different consonant sounds at the beginning of words. Specific tests for autism, the Autism Diagnostic Observation Schedule (ADOS) and the Childhood Autism Rating Scale (CARS), were administered. The test results were as follows: the CARS score was 30 (a score of ≥30 indicates mild to moderate autism symptoms), and the ADOS communication score was 6 and social interaction score was 10, both meeting the autism diagnosis. Additionally, the patient frequently presented with refractory eczema and wheezy bronchitis due to the allergic constitution. The results of allergen detection reported allergies to egg (++, should be avoided), milk (+++, should be avoided), and house dust (++, should be avoided). The total blood IgE and lgA were 355.7 IU/mL (<52 IU/mL) and 0.34 g/L (0.70–4.00), respectively. The patient had a history of previous immune thrombocytopenic purpura.

At age 3 years, the patient is capable of running, but struggles with unbuttoning clothing and putting on shoes. In addition, the patient is unable to form simple two-to-three-word sentences to express their needs and desires. Listeners familiar with the patient may have difficulty understanding their speech most of the time. The patient is also unable to request or draw attention to objects by verbally identifying them. According to language development assessment, language comprehension reached only the level of 1.5–2 years, and language expression reached only the level of 1.5–2 years. His ability to execute instructions and social skills were also weak. Besides, he had weak cognitive and spatial conceptual abilities (e.g., superior-inferior, anterior-posterior, and left-right), and he was unable to identify among colors and shapes. The intelligence score was 69. Assessment for autism reported hyperactivity, inattention, and temporary eye contact. Physical examination reported wandering testes and genu valgus. Cranial MRI revealed insufficient myelination of cerebellar white matter (Figures 2B, C).

Family history: the patient’s parents were unrelated. The father had mild intellectual disability, poor thinking, poor ability to communicate, poor expressive and receptive language skills, grammatical impairment, tone and behavioral abnormalities. He was obese as a child but had a normal body shape now. The patient’s grandparents, mother and older brother were normal. Unfortunately, the data on the immune abnormalities of proband’s father is not available.

Treatment included mainly exercise therapy, sensory integration training, language training, acupuncture, etc.

## Genetic detection and analysis

**FIGURE 3:**
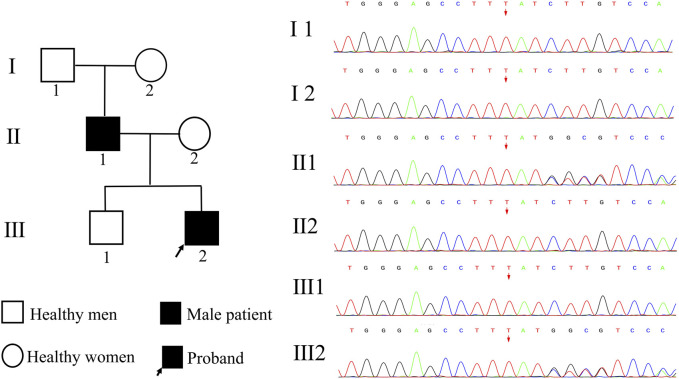
Pedigree map and genetic test results. I 1 represents the proband‘s grandfather, I 2 represents the proband’s grandmother, II 1 represents the proband‘s father, II 2 represents the proband’s mother, III 1 represents the proband‘s brother, and III 2 represents the proband. Both the proband and his father carried a heterozygous variant of TCF20, c.18391872del, and the remaining family members were wild-type.

We followed the materials and methods outlined as follows: 2 ml of peripheral blood was collected from the proband, his parents, brother, grandfather, and grandmother. The genomic DNA of peripheral blood cells was extracted using a DNA extraction kit, and Illumina Hiseq X Ten was chosen for whole exome sequencing. The average sequencing depth achieved was approximately 150×, with 99.90% coverage of the target region. Sequences were compared utilizing Burrows Wheeler Alignment tool software, with reference genome sequence GRCh37. GATK was employed to rearrange sequences and identify, annotate, and statistically analyze variants. Sanger sequencing was conducted on potential pathogenic variants to analyze their sources, and data interpretation rules followed the guidelines provided by the American College of Medical Genetics and Genomics (ACMG). A heterozygous variation, c.1839_1872del (p.Met613IlefsTer159), was detected in the *TCF20* in the patient. This variation caused nucleotide deletion at positions 1,839-1,872, leading to conversion from methionine to isoleucine at position 613 and a downstream premature termination codon at position 159, consistent with frameshift variation. According to Sanger sequencing, the variation was derived from the father, in line with the autosomal dominant pattern of inheritance. Co-segregation between genotype and clinical phenotype in this family was demonstrated (Figure 3).

The *TCF20* variation c.1839_1872del (transcript: NM_000158) is not included in multiple variant databases (1000G, ESP6500, ExAC, gnomAD) and diseases databases (HGMD, ClinVar), and it was identified as a nonpolymorphic variation.

**FIGURE 4:**
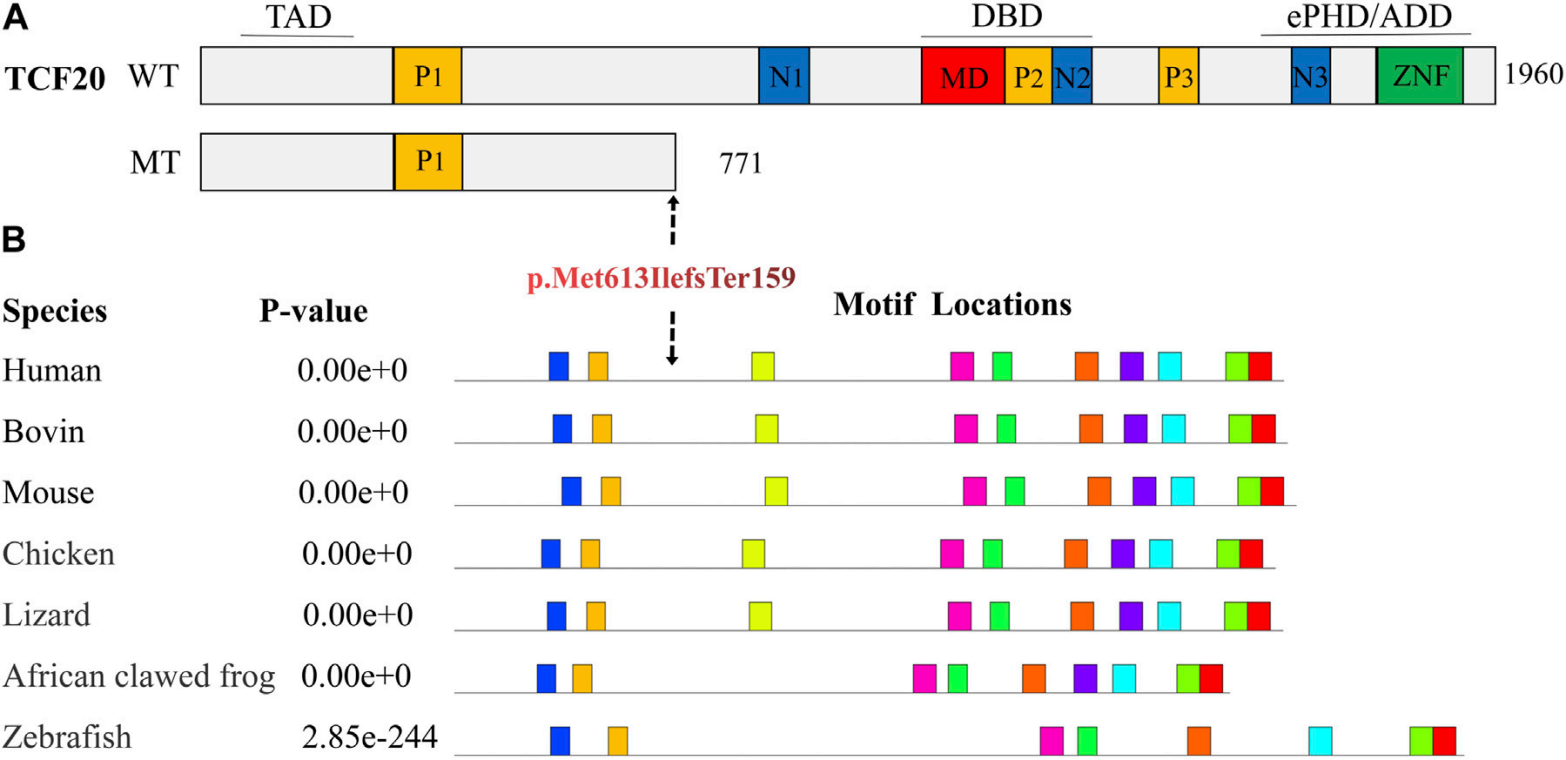
Prediction of conserved functional domains of TCF20. **(A)**. Diagram representing the TCF20 with previously annotated domains: P1-P3, PEST domains; N1-N3, nuclear localization signals; MD, minimal DNA binding domain; ZNF, zinc finger domain. The three lines above the protein denote the following domains: TAD, transactivation domain; DBD, DNA binding domain and the ePHD/ADD domain. WT indicates wild type; MT indicates mutant type, p. Met613IlefsTer159, and TCF20 that produces truncations after variation loses critical domains, including nuclear localization sequences, AT-hook, DNA-binding domains, PHD domains, zinc finger domains, etc. **(B)**. shows the predicted conserved domains, and the amino acid sequence of TCF20 is highly conserved in Human, Bovin, Mouse, Chicken, Lizard, African clawed frog, and Zebrafish, which will lead to the loss of a large number of conserved functional domains after mutation.

Analysis of conserved amino acid sequences was performed using the online software MEME. The result showed that the amino acid sequences of the *TCF20* were highly conserved in Human, Bovin, Mouse, Chicken, Lizard, African clawed frog and Zebrafish (Figure 4). After a search on the bioinformatics database MutationTaster, the frameshift variation c.1839_1872del caused p. Met613IlefsTer159, leading to changes in the amino acid sequences after position 613 and loss of a large segment of conversed amino acid sequences (motif 3–8) due to production of truncated proteins. As a consequence, the haploinsufficiency thereafter changed the structure and function of the proteins. The frameshift variation c.1839_1872del was identified as 100% pathogenic.

Based on the 2020 American College of Medical Genetics and Genomics (ACMG) criteria, c.1839_1872del in *TCF20*, in line with PVS1, PM1, PM2, PM4, PP1 and PP3, is classified as a pathogenic variation.

## Discussion

TCF20 is widely expressed in various tissues, and its variation can lead to clinical symptoms that have significant heterogeneity. Developmental disorder of the nervous system is the common, predominant clinical manifestation, and there may have some concomitant congenital malformation of other systems (Torti et al., 2019; Vetrini et al., 2019). The patient in this report presented with mainly developmental disorders of the nervous system, including intellectual impairment, retarded motor and speech development, reduced muscle strength and tension, and increased pain threshold, accompanying multisystem abnormalities such as obesity, special appearance, and testicular descent. In addition, this patient also developed rare symptoms of the immune system, including hyper-IgE, immune thrombocytopenic purpura, cows milk protein allergy, and wheezy bronchitis.

Gene sequencing identified a heterozygous variation in the *TCF20*, c.1839_1872del (p.Met613IlefsTer159), in the patient. This variation may lead to loss of key domains (e.g., nuclear localization sequence, AT-hook, DNA-binding domain, PHD domain and zinc-finger domain) in the truncated protein or nonsense-mediated RNA degradation due to sequence variation, affecting the nuclear localization of TCF20. Bioinformatics analysis demonstrated higher intolerance of *TCF20* to loss-of-function variants as reflected by its higher pLI (probability of loss-of-function intolerance) score (pLI = 1) (Vetrini et al., 2019). In addition, TCF20 is a transcriptor co-activator that can enhance the activity of multiple transcription factors (e.g., JUN, SP1, PAX6, ETS1, MDC1, QRICH1, RNF4, ANDR, AR, GATAD2B, PHF14, SIN3A, P66B and RAI1) to play a role in various physiological processes, such as DNA damage repair, epigenetic regulation of gene expression, protein modification, signal transduction, cellular proliferation, differentiation and apoptosis, neuronal differentiation, synaptic development, genito-urinary system development, immune response and lipid metabolism. Loss of key domains in the TCF20 affects the activity of other related transcription factors (Rekdal et al., 2000).

In early embryos, TCF20 has implications for the development and function of the nervous system by involving the neuronal proliferation and differentiation under multiple mechanisms. The latest research demonstrated that TCF20 regulated the expression of DNA demethylation factor TGD and subsequently the level of DNA methylation in the promotor region of the TCF-4 gene associated with neural differentiation, thereby modulating neuronal differentiation and nervous system development. It was also reported that loss of TCF20 gene significantly reduced the number of neurons, resulting in abnormalities of brain function (Feng et al., 2020). Another study revealed that TCF20 was highly co-expressed with MeCP2 in neurons, and they cooperated to regulate the expression of key neuronal genes. Experimental results confirmed that disruption of the TCF20-MeCP2 complex was the basis of NDD. In addition, that study also found that the structural disruption and loss of function of TCF20 protein due to gene variation led to poor cooperation with MeCP2, inducing NDD (Zhou et al., 2022). Moreover, TCF20 can also act on estrogen (ER) and androgen (AR) receptors to play a role in circadian rhythm, and changes in TCF20 activity may be linked with sleep disorder, hyperactivity, anxiety, obsessive-compulsive disorder, and overgrowth, etc (Vetrini et al., 2019).

The patient in this report carried a pathogenic variation in the *TCF20* and then developed rare immune system disorders. Presently, the specific mechanism by which TCF20 variation induces immune system diseases has not yet been reported. *TCF20* also shows wide expression in immune cells. As a transcriptor co-activator, TCF20 can regulate not only the proliferation and differentiation of immune cells, but also the activity of transcription factors involved in the regulation of adaptive and acquired immune response (e.g., RNF4, SIN3A, FBXL19, ETS1, SP1 and JUN). TCF20 and RAI1 are paralogous, stemming from a gene duplication event early in vertebrate evolution. They share 45% overall similarity with 7 domains sharing 97% sequence similarity. In addition, they possess similar evolutionarily conserved functional domains, including N-terminal trans-activating domain, NLS, and zinc-finger domain, etc. The two proteins are highly similar to each other in both structure and function (Babbs et al., 2014; Pounraja and Girirajan, 2019; Vetrini et al., 2019; Lévy et al., 2022). RAI1 can interact with TCF20 via the zinc-finger domain (Babbs et al., 2014). An animal experiment in mice with allergic rhinitis showed that RAI1 could influence Th1/Th2 balance and regulate the release of IgE by plasma cells (Yang and Xiao, 2019). Another study found that RAI1 was a negative regulator of the survival and activation of lymphocytes, and loss of RAI1 protein led to the breakdown of immunological tolerance while development of systemic autoimmunity (Savino et al., 2009). It was also reported that loss of TCF20 function might lead to immune system diseases, such as autoimmune hepatitis and hyper-IgE (Torti et al., 2019).

The patient and his father carried an identical variation in the *TCF20*, but their phenotypes were different. Irregular dominance was demonstrated. Similarly, Vetrini et al. (2019) reported varying phenotypes between monozygotic twins with an identical *TCF20* variant. In a longitudinal study on cognitive characteristics and functions (Mustafin et al., 2020), the phenotype induced by *TCF20* variation was affected by epigenetic effects and closely associated with the environmental factors including nutrition, pre- and post-natal stress. In addition, this study noted that each individual had unique epigenetic response to environmental stimuli, resulting in variable penetrance, variable presence of penetrance and expressivity. Moreover, such response varied with age. Combining the studies, the diseases induced by TCF20 variation may not be typical monogenic diseases, instead, they may be a result of interplay between multiple genetic and environment factors. In most cases, TCF20 variation-induced diseases are spontaneous that can be autosomal dominant (Torti et al., 2019). Such disease has a 50% probability of being present in offspring of the patient or another child of the parents. Since the site of variation has been identified, amniocentesis and prenatal diagnosis can be performed during pregnancy to avoid the incidence of such disease in future clinical practice.

Currently, there is no effective therapy available to treat TCF20-related diseases, and prevention becomes the focus. We provided language, cognition, and social communication rehabilitation training to the boy. By the time they reached 3 years and 1 month of age, he showed more interest in their surroundings. He was able to initiate social interaction, and his motor imitation and play skills had significantly improved compared to before. The score on the CARS review decreased to 22 points, while the adaptability score on the Gesell assessment increased to 70.2 points (equivalent to a developmental age of 26 months) and the social communication score increased to 69.1 points (equivalent to a developmental age of 25.6 months), all of which indicated significant improvement in the children’s symptoms of autism. Previous literature reported that *TCF20* variation is associated with some severe neuropsychiatric disorders (e.g., schizophrenia, generalized anxiety disorder and seizure (Smeland et al., 2017; Vetrini et al., 2019; Lévy et al., 2022)) and the occurrence, development, metastasis and recurrence of multiple tumors (e.g., gliomas, meningiomas, and ovarian and colon cancers (Rekdal et al., 2000)). Given the delayed penetrance, persistent follow-up of these phenotypes is warranted.

## Data Availability

The datasets for this article are not publicly available due to concerns regarding participant/patient anonymity. Requests to access the datasets should be directed to the corresponding author.
